# Induction of innate cytokine responses by respiratory mucosal challenge with R848 in zebrafish, mice, and humans

**DOI:** 10.1016/j.jaci.2019.04.003

**Published:** 2019-07

**Authors:** Fränze Progatzky, Akhilesh Jha, Madina Wane, Ryan S. Thwaites, Spyridon Makris, Robin J. Shattock, Cecilia Johansson, Peter J. Openshaw, Laurence Bugeon, Trevor T. Hansel, Margaret J. Dallman

**Affiliations:** a Department of Life Sciences, Faculty of Natural Sciences, Imperial College London, London, United Kingdom; b National Heart and Lung Institute, Imperial Clinical Respiratory Research Unit (ICRRU) and Respiratory Infection, St Mary's Hospital, Imperial College London, London, United Kingdom; c Department of Infectious Diseases, Division of Medicine, Imperial College London, London, United Kingdom

To the Editor:

Development of new therapies and vaccines to combat viral respiratory tract infections is slow, partly because of the limited understanding of innate immune responses at the respiratory mucosal site of disease. Detailed characterization of such responses might facilitate biomarker definition for respiratory diseases and provide novel mechanistic insights and a platform for the testing of novel therapeutics. Recently, noninvasive serial nasosorption of mucosal lining fluid has been used to study immune responses to experimental live human rhinovirus.^1^ However, human viral infection models require specialized centers and resources, with some studies requiring quarantine of volunteers.

*In vivo* animal models of innate immune stimulation are useful alternatives; for example, mammalian models of airway mucosal polyinosinic:polycytidylic acid (poly[I:C]) challenge (a viral double-stranded RNA mimetic) are well established and demonstrate the ability of these agents to induce proinflammatory cytokines by respiratory cells.^2^, ^3^, ^4^ Many noninfectious models of innate antiviral immunity have used resiquimod (R848; a Toll-like receptor [TLR] 7/8 agonist, single-stranded RNA mimetic), which is closely related to imiquimod. R848 causes different vaccine-specific immune responses in minipigs when administered intradermally or intranasally, while intranasal R848 had adjuvant activity in macaques.^5^ Studies using these models in mice, chimpanzees, and ferrets have provided valuable insight into the mechanisms of immunity to and pathogenesis of viral respiratory tract infections. However, they are not always practical to use, and they do not always accurately mimic human infection responses. Furthermore, the extent to which these models predict human vaccine efficacy is often unclear.^6^, ^7^

The zebrafish *(Danio rerio)* is an attractive alternative vertebrate species, especially because of similarities with the human innate and adaptive immune system. Recently, we have used zebrafish gills to assess respiratory inflammation, and our results suggest zebrafish are a relevant model to study mechanisms of respiratory mucosal innate immune responses.^8^ Therefore, we developed parallel live zebrafish, mouse, and human challenge models to study the effects of viral RNA mimic TLR agonists with relevance to respiratory viral infection. These comparative studies allow assessment of cytokine responses at comparable and accessible sites of the respiratory mucosa (Fig 1, *A-C*).

**Fig 1:**
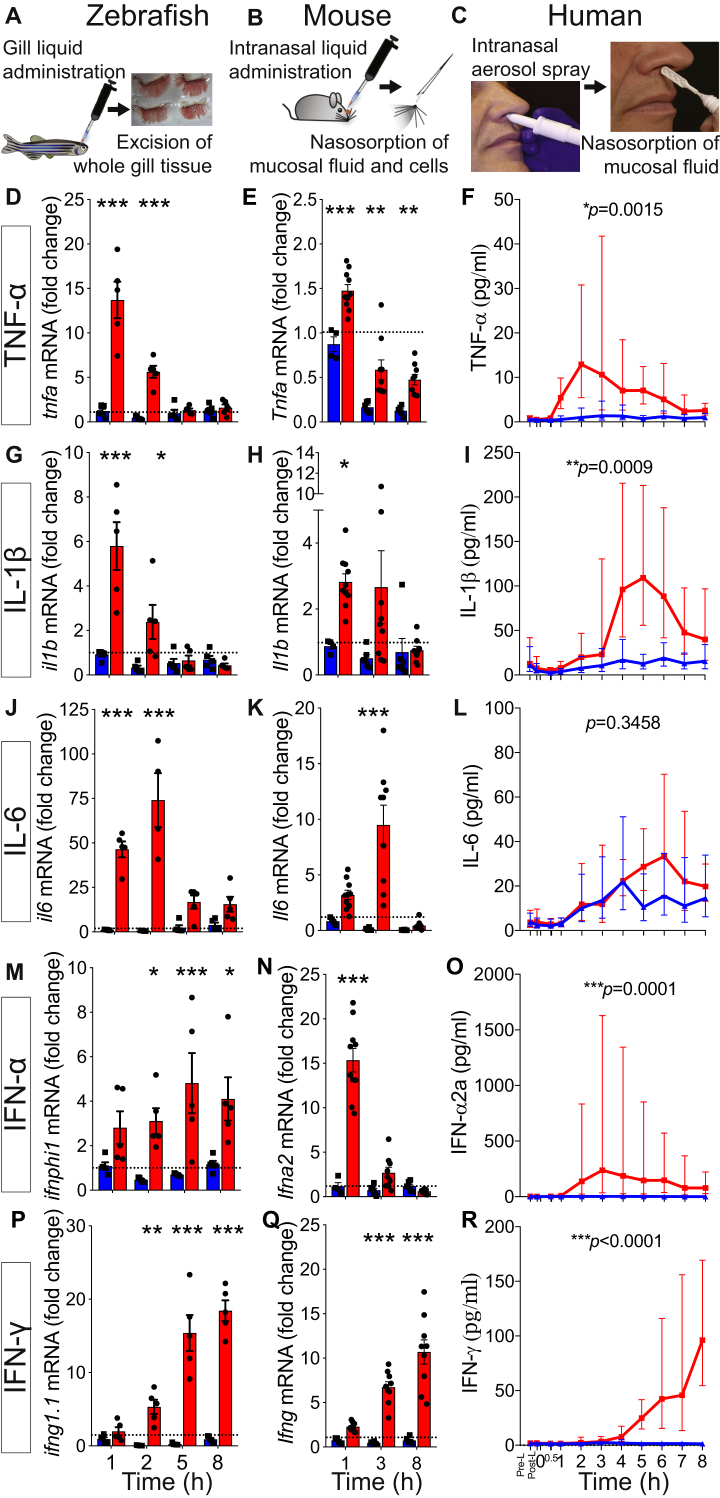
Kinetic profile of mucosal proinflammatory cytokine responses after zebrafish gill, mouse, and human nasal stimulation with R848. **A-C,** Schematics showing mucosal administration of TLR agonists and sampling of mucosal tissue/fluids to assess responses. **D, G, J, M,** and **P,** qRT-PCR analysis of zebrafish gills (n = 5, representative of 3 experiments). Values are presented as means ± SEMs. Two-way ANOVA followed by the Sidak multiple comparison test was used. **E, H, K, N,** and **Q,** qRT-PCR analysis of mouse nasal mucosa (n = 4-10 pooled from 2 independent experiments). Values are presented as means ± SEMs. Two-way ANOVA followed by the Sidak multiple comparison test was used. **F, I, L, O,** and **R,** Soluble protein mediator analysis of human nasal samples (n = 9). Values are presented as geometric means and 95% CIs. Paired *t* tests on log_10_-transformed area under the curve values were used. *P < .05, **P < .01, and ***P < .001.

Human nasal samples were collected serially by means of nasosorption (using a synthetic absorptive matrix [SAM]) after saline and TLR agonist nasal challenge of 9 volunteers (see Table E1 in this article's Online Repository at www.jacionline.org for baseline characteristics of participants and Table E2 in this article's Online Repository at www.jacionline.org for nasal and systemic observations and clinical symptoms after R848 administration). The mouse nasal cavity is inaccessible for repetitive sampling, and therefore we developed a mucosal tissue sampling technique *ex vivo* by applying an absorption approach similar to that used for human subjects.

Zebrafish whole gill tissue was harvested at several similar time points after R848 challenge. Fig 1 shows how remarkably similar cytokine responses were across the 3 species, especially between zebrafish and human subjects (see Fig E1 in this article's Online Repository at www.jacionline.org for detailed responses of individual human subjects). An early response was observed for TNF-α, whereas IFN-γ levels increased later. These results suggest that R848 can be administered to human subjects as a noninfectious virus-type challenge of the innate immune system, whereas complementary studies in mice and zebrafish could allow mechanistic insight.

When poly(I:C) was applied, neither the fish gill nor human nose responded (see Fig E2 in this article's Online Repository at www.jacionline.org). In contrast, the mouse nasal mucosal response to poly(I:C) was characterized by an early increase in *Tnfa*, *Il6*, and *Ifna2* transcript levels and a later increase in *Ifng* transcript levels. Overall, the R848 and poly(I:C) challenges demonstrated both matching and discrepant innate antiviral responses in the different models.

To further refine the use of zebrafish gills as a model to study viral mimetics, we also established a noninvasive sampling technique using SAM to allow for repetitive sampling and thereby longitudinal studies of individual fish, which also contributes to the 3Rs (replacement, reduction, and refinement) through refinement and reduction of animal procedures. *Tnfa*, *il1b*, *ifnphi1*, and *ifng1.1* transcripts were successfully detected by using this method and significantly increased in gills stimulated by R848 (see Fig E3, *A-D*, in this article's Online Repository at www.jacionline.org). Making use of transgenic zebrafish with labeled immune cells, we examined both neutrophil *(Tg[lyz:GFP])* and lymphocyte *(Tg[lck:eGFP])* distribution within the gill tissue and found a significant early transient increase in neutrophil counts (Fig 2, *A* and *B*), followed by an increase in lymphocyte counts (Fig 2, *C* and *D*) in the lamella after R848 stimulation. Such cells, but not vascular epithelial cells, were also harvested by using SAM (see Fig E3, *E-I*). These data highlight the number of useful features of the zebrafish respiratory mucosal model that permit investigation of mechanistic immune pathways for assessing topical drug effects, viral infections, and vaccine adjuvant activity.

**Fig 2:**
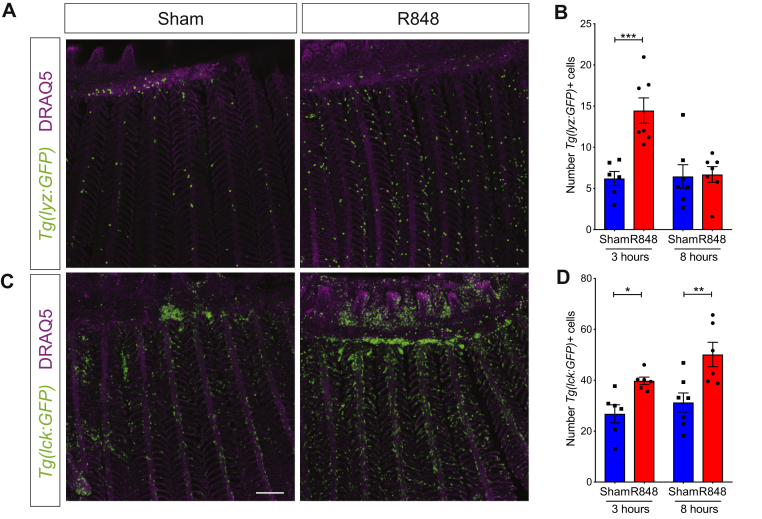
R848 induces lymphocyte migration in zebrafish gills. **A** and **C,** Maximum z-stack projections of *Tg*(*lyz:GFP*; neutrophils; Fig 2, *A*) and *Tg*(*lck:GFP*; lymphocytes; Fig 2, *C*) gills after treatment with water or R848 for 3 (Fig 2, *A*) and 8 (Fig 2, *C*) hours (n = 7). *Scale bars* = 100 μm. **B** and **D,** Average number of GFP^+^ cells in the first 20 lamellae of each filament. Each *dot* indicates average counts per individual fish (n ≥ 6). Values are presented as means ± SEMs. Two-way ANOVA followed by the Sidak multiple comparison test was used.

Animal models are central to our understanding of innate antiviral immunity. However, translation of these studies to human disease can be limited. This can result in the need for primate models of disease that are ethically, financially, and logistically challenging. Here we establish parallel methods for administration of TLR ligands directly onto the respiratory mucosa in 3 species, with measurement of local inflammation using simple and reproducible sampling methods. Development of a human nasal mucosal model is of special interest because the nose is the portal for viral respiratory tract infections that cause widespread winter morbidity and mortality, and there are advantages in studying a complex multicellular mucosal system directly in human subjects.

Using these approaches, we demonstrate remarkably analogous interferon and inflammatory cytokine production after R848 stimulation in the human and mouse nasal mucosa and zebrafish gill tissue, which was evident despite the functional inactivity of mouse TLR8.^9^ By contrast, human subjects and zebrafish did not respond to poly(I:C), demonstrating that this common mouse model of viral innate immune activation might have limited translation to the human mucosal response to double-stranded RNA viruses.

This study demonstrates that respiratory challenge with R848 might offer a novel mucosal model of antiviral immunity in human subjects. This human challenge model might be particularly suited to understanding differences in innate antiviral responses in patients with allergic and respiratory diseases, such as asthma and chronic obstructive pulmonary disease, in which viral infections are major exacerbation triggers. As such, mechanistic studies using poly(I:C) challenge of mice might lack direct translation to human subjects. Instead, the R848 model can confidently be extended to mice and zebrafish, in which the analogous response to R848 allows more detailed mechanistic insights, relatable to those seen in human subjects. Overall, these novel parallel *in vivo* mucosal models offer a platform for translational studies and trials of novel antiviral therapies, vaccines, and adjuvants.

## Figures and Tables

**Fig E1:**
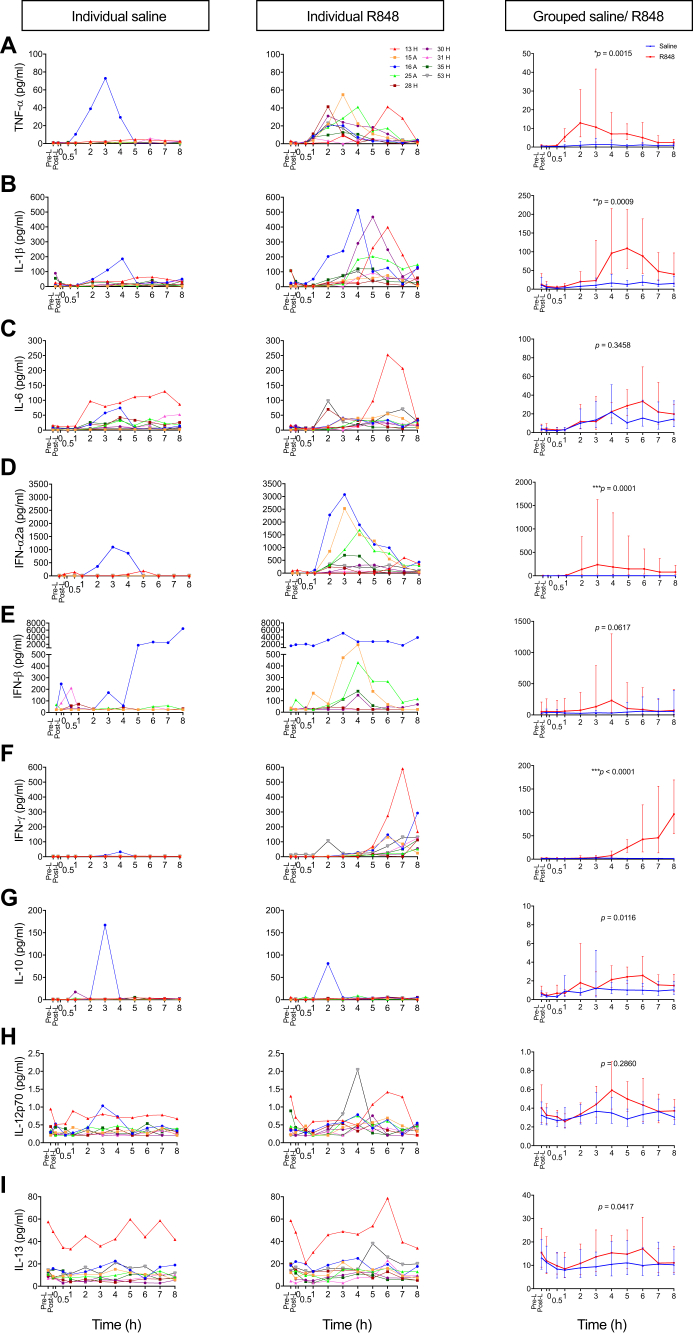
Kinetic immune response of nasal mediators induced by R848 nasal challenge in individual subjects. **A-I,** Immune response of individual subjects (n = 9) after saline *(left panels)* and R848 *(middle panels)* challenge. Of interest, the 3 volunteers with atopy had relatively enhanced type 1 interferon responses, although this study was not specifically powered to address this question. Grouped data *(right panels)* are displayed as geometric means and 95% CIs. *Blue line*, Saline; *red line*, R848. Paired *t* test on AUC of log_10_-transformed values between 0 and 8 hours in individual subjects after nasal challenge was used. IFN-β values: n = 7. *A*, Volunteer with allergic rhinitis; *H*, healthy.

**Fig E2:**
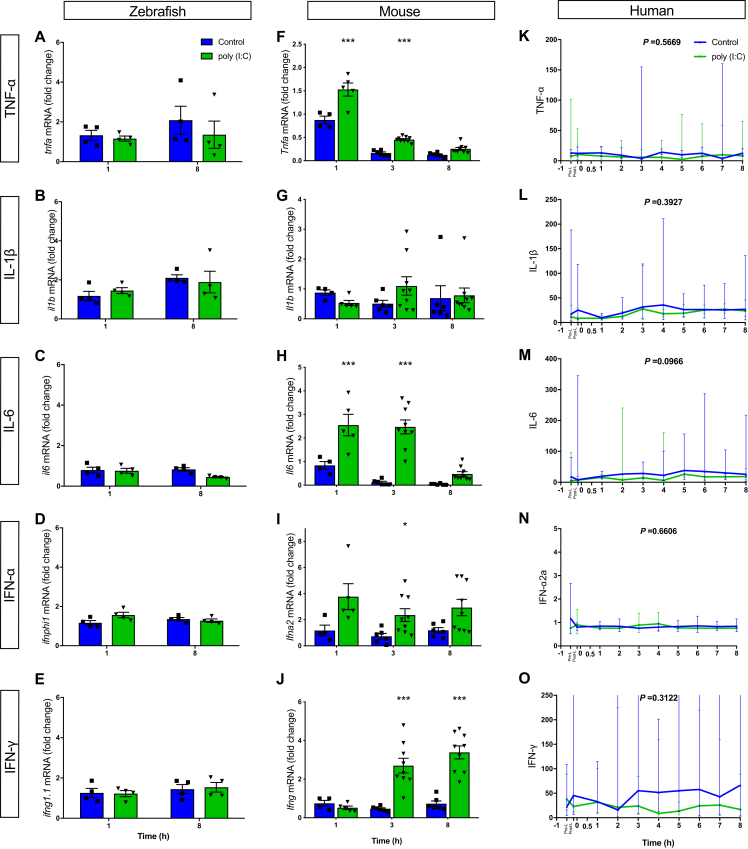
Kinetic profile of mucosal proinflammatory cytokine responses after zebrafish gill, mouse, and human nasal stimulation with poly(I:C). qRT-PCR analysis of *tnfa***(A)**, *il1b***(B)**, *il6***(C)**, *ifnphi1***(D)**, and *ifng1.1***(E)** transcripts in zebrafish gills (n = 4, representative of 2 experiments) after gill treatment. Values are presented as means ± SEMs. Two-way ANOVA followed by the Sidak multiple comparison test was used. qRT-PCR analysis of *Tnfa***(F)**, *Il1b***(G)**, *Il6***(H)**, *Ifna2***(I)**, and *Ifng***(J)** transcripts in mouse nasal mucosa (n = 4-10 pooled from 2 independent experiments) sampled with SAM after intranasal stimulation. Values are presented as means ± SEMs. Two-way ANOVA followed by the Sidak multiple comparison test was used. Soluble protein mediator analysis of TNF-α **(K)**, IL-1β **(L)**, IL-6 **(M)**, IFN-α2α **(N)**, and IFN-γ **(O)** in human nasal mucosal lining fluid (n = 8), as measured by using the nasosorption technique after challenge. Values are presented as geometric means and 95% CIs, and AUC analyses were used. *P < .05 and ***P < .001.

**Fig E3:**
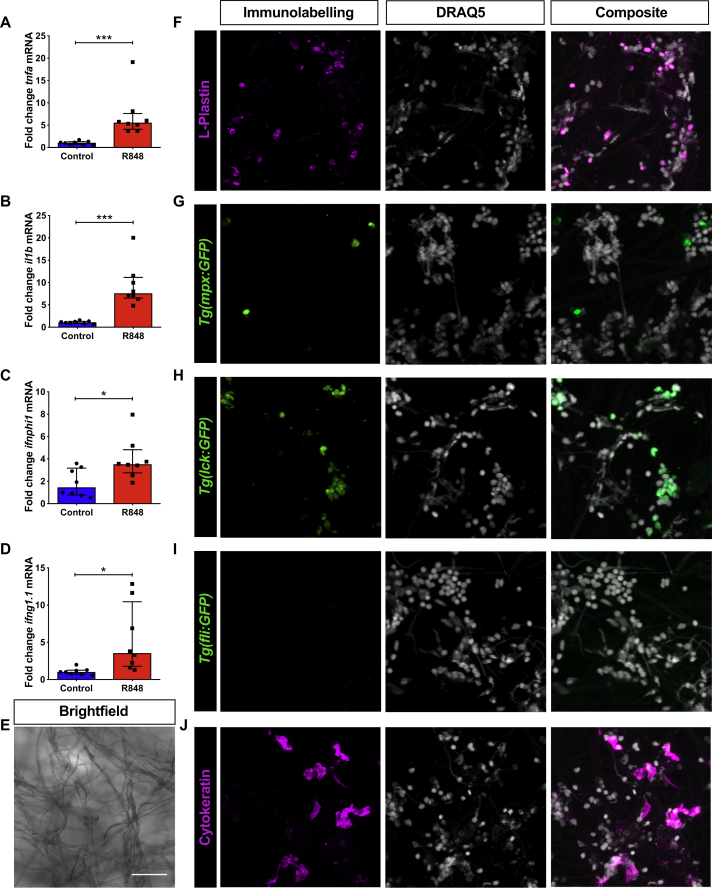
Gillsorption as a noninvasive sampling tool to monitor mucosal proinflammatory cytokine and interferon responses after zebrafish gill stimulation with R848. **A-D,** qRT-PCR analysis of adult zebrafish gills sampled with SAM 1 hour after gill treatment with water (control, *blue bars*) or R848 (0.5 mg/mL, *red bars*) for 5 minutes. *Dot plots* show relative expression values obtained for individual fish (n = 8), which were normalized to 18S and expressed as fold change relative to the median control sample. Values are presented as means ± SEMs. *P < .05 and ***P < .001, Mann-Whitney test. **E,** Representative brightfield image of SAM corresponding to Fig E3, *J*. **F-J,** Representative images (maximum projection of confocal *z*-stack) of cells absorbed by SAM after application on gill tissue of live WT zebrafish stained with an L-plastin antibody (red; Fig E3, *F*) or the transgenic zebrafish *Tg(mpx:GFP)* (Fig E3, *G*), *Tg(lck:GFP)* (Fig E3, *H*), and *Tg(fli:GFP)* (Fig E3, *I*) stained with an anti-GFP antibody (green) or a cytokeratin antibody (red; Fig E3, *J*). SAM was costained with DRAQ5 (cyan).

**Table E1:** Baseline characteristics of participants undergoing R848 nasal challenge

Subject code	Age (y)	Sex	Positive grass pollen skin prick test response?	Eosinophil count (× 10^9^/L)	Serum total IgE
13	45	Male	No	0	23.5
15	29	Female	Yes	0.1	67.5
16	49	Female	Yes	0.2	1085
25	35	Female	Yes	0.2	142
28	34	Female	No	0.3	54.1
30	27	Female	No	0	32.8
31	47	Female	No	0	4.69
35	45	Female	No	0.1	NA
53	32	Female	No	NA	2.12

*NA*, Data not available.

**Table E2:** Nasal and systemic symptoms after R848 nasal challenge

Subject code	Nasal symptoms	Fever >37.2°C	Myalgia	Fatigue	Headache	Comments
13	Nil	No	No	No	No	Asymptomatic
15	Nil	No	Yes	Yes	Yes	Fatigue for up to 36 h after challenge
16	Blocked nose	Yes	No	No	No	Nose blocked at 3 h after challenge for 1 h Temperature increase from 36.9°C to 37.4°C Asymptomatic
25	Blocked + itchy nose	Yes	Yes	Yes	Yes	Shivering soon after administration of R848 for 1.5 h Flu-like symptoms overnight with nasal symptoms
28	Nil	No	No	No	No	Asymptomatic
30	Nil	No	No	No	No	Asymptomatic
31	Nil	No	No	No	No	Asymptomatic
35	Nil	No	No	No	No	Asymptomatic
53	Nil	Yes	Yes	Yes	Yes	Flu-like symptoms for 24 h after challenge Temperature increased to 39.2°C Mild-to-moderate headache

## Methods

### Human studies

#### Participants

This human study was approved by the London-Harrow Research Ethics Committee (clinicaltrials.gov identifier NCT02090374). Healthy participants were enlisted from a database of previous volunteers, as well as recruitment through newspaper advertisements. Subjects were aged between 18 and 60 years and were current nonsmokers with a history of less than 5 pack years. Participants had no history of asthma, respiratory illness, or any other major systemic disease and were required to be off nasal, inhaled, and systemic steroids. Participants with allergic rhinitis were permitted. Written informed consent was obtained from all volunteers before enrollment.

#### Study design

The study consisted of 2 nasal challenges: first with saline and then followed 1 to 4 weeks later by a TLR agonist (either R848 or poly[I:C]). On the day of the challenge visit, nasal mucosal lining fluid was sampled at baseline by using nasosorption (Nasosorption FX-i; Hunt Developments, Midhurst, United Kingdom) for a duration of 1 minute before and after nasal lavage, as described previously.^E1^ After nasal challenge, nasosorption was performed at 30 minutes and then at hourly intervals for up to 8 hours in all subjects. Clinical parameters of nasal obstruction were assessed by using Total Nasal Symptom Scores and peak nasal inspiratory flow, as described elsewhere.^E2^

#### Nasal challenge with TLR agonists

TLR agonists used in the study included high-molecular-weight poly(I:C) from InvivoGen (San Diego, Calif) and VacciGrade R848 (also from InvivoGen). Saline and TLR agonists were administered by using a Bidose nasal delivery device (Thermo Fisher, Epsom, United Kingdom), which emits 100 μL per actuation. Poly(I:C) has been previously tolerated in another study at doses of up to 800 μg per nostril over a 24-hour period, with no adverse events reported.^E3^ Therefore poly(I:C) was administered at a dose of 500 μg per nostril in five 100-μL installments by using the Bidose over a 1-hour period. R848 was delivered at a dose of 10 μg per nostril using 1 actuation of the Bidose device.

#### Sample processing

Mucosal lining fluid obtained from volunteers was eluted, as described previously,^E1^ and stored in assay buffer at −80°C, in addition to culture supernatants from PBMC experiments. These were thawed out before chemokine and cytokine assays, which were performed by using the MSD platform (Mesoscale Diagnostics, Gaithersburg, Md) with proinflammatory panel 1 (IFN-γ, IL-10, IL-12p70, IL-13, IL-1β, IL-2, IL-4, IL-6, IL-8, and TNF-α) and an IFNα2a kit.

### Mouse studies

#### Mice

C57BL/6 mice were purchased from Charles River or Harlan Laboratories (United Kingdom). All mice were maintained in pathogen-free conditions, and sex- and age-matched mice aged 8 to 10 weeks were used for each experiment. All animal experiments were reviewed and approved by the Animal Welfare and Ethical Review Board within Imperial College London and approved by the UK Home Office in accordance with the Animals (Scientific procedures) Act 1986 and the ARRIVE guidelines.

#### Intranasal stimulation with TLR agonists, mouse nasosorption, and quantitative RT-PCR

For exposure to TLR ligands, mice were lightly anesthetized and instilled intranasally with poly(I:C) high molecular weight or resiquimod (R848: VacciGrade), both from InvivoGen, at 2.5 μg per gram of mouse weight in 100 μL (= 50 μg/100 μL per mouse). Mice were killed at 1, 3, and 8 hours after exposure by means of intraperitoneal administration of pentobarbital.

To gain access to the nasal cavity, the snout and cartilage were removed with scissors. To sample the mucosa, a small piece of SAM was inserted into both nostrils by using fine tweezers. RNA from the SAM was extracted by using TRIzol and the PureLink RNA Micro Kit (Life Technologies, Grand Island, NY), according to the manufacturer's instructions. cDNA was synthesized from 500 ng of total RNA, and quantitative RT-PCR (qRT-PCR) was carried out, as described below. The following TaqMan primers and probes were used: *Tnfa* (Mm00443260_g1), *Il1b* (Mm00434228_m1), *Il6* (Mm_00446190_m1), *Ifng* (Mm00801778_m1), *Ifna2* (Mm00833961_s1), *Ifnb1* (Mm00439546_s1), and *Hprt* (Mm00446968_m1). Cycle threshold (Ct) values obtained were normalized to *Hprt* and calibrated to the median control sample for relative quantification by using the comparative Ct method.

### Zebrafish studies

#### Zebrafish care

Wild-type zebrafish used in this study were reared and maintained according to standard practices at 28.5°C on a 14-hour light/10-hour dark cycle. All procedures conformed to UK Home Office requirements (ASPA 1986). The transgenic zebrafish lines *Tg(lyz:GFP)*,^E4^ *Tg(mpx:GFP)*, (*Tg(mpx:GFP)i114*),^E5^ *Tg(lck:GFP)*,^E6^ and *Tg(fli:GFP)*^E7^ were used.

#### Zebrafish gill treatment with TLR ligands and gillsorption

Zebrafish were anesthetized in 0.17 mg/mL tricaine methanesulfonate (MS-222; Sigma, St Louis, Mo) solution and positioned laterally under a dissecting microscope on a Petri dish. Any water was carefully removed, and the right gill side of the fish was gently dabbed with tissue. R848 (2.5 μg/5 μL per zebrafish, InvivoGen) or poly(I:C) (10 μg/5 μL per zebrafish, Invivogen) was applied unilaterally to the gill for 5 minutes. Fish were returned to a fresh system water.

To analyze transcript changes after stimulation, zebrafish were either killed in MS-222 (4 mg/mL) and their gills were dissected or they were anesthetized in MS-222 (0.17 mg/mL) and their gill tissues were gently dabbed to remove excess water, with a piece of SAM (Fibrous Polyester; Hunt Developments)^E1^ applied for 5 seconds before being returned to fresh system water.

#### RNA extraction, cDNA synthesis, and qRT-PCR

Dissected gill tissue from adult zebrafish were homogenized with a pestle in lysis buffer and processed for RNA by using the MagMAX-96 Total RNA Isolation Kit (Thermo Fisher) or the PureLink RNA Micro Kit (Thermo Fisher), according to the manufacturer's instructions. To extract RNA from cells attached to SAM after gillsorption, the SAM was placed in TRIzol and then separated from the TRIzol by means of centrifugation with Corning Costar Spin-X centrifuge tube filters (without membrane), followed by RNA processing with the PureLink RNA Mini Kit (Life Technologies), according to the manufacturer's instructions. The quantity and quality of RNA were assessed spectrophotometrically by using a Thermo Scientific NanoDrop 1000. One hundred twenty-five nanograms of total RNA was used for reverse transcription by using a High-Capacity cDNA Archive Kit (Applied Biosystems, Foster City, Calif), according to the manufacturer's instructions. qRT–PCR was performed with 2% cDNA generated by using TaqMan Fast Universal 2× PCR Master Mix (Applied Biosystems) and TaqMan primer and probe assays for 18S (4319413E), zebrafish *Il1b* (Dr03114368_m1), *Tnfa* (Dr03126850_m1), *Il6* (FAM/MGB-NFQ custom TaqMan RNA assay: TGGAGGCCATAAACAGCCAGCTGCA), *Ifnphi1* (Dr03100938_m1), and *Ifng1.1* (Dr03109489_g1). All reactions were performed in duplicates by using a 7500 Fast Real-time PCR system (Applied Biosystems). Ct values obtained were normalized to 18S and calibrated to the median control sample for relative quantification by using the comparative Ct method.

#### Whole-mount immunostaining

Dissected gills were fixed in 4% paraformaldehyde at 4°C overnight. Gills were washed twice in PBS and once in deionized water, incubated in acetone at −20°C for 10 minutes, and rinsed in deionized water and twice in PBS-T (PBS, 0.05% Triton X, and 0.05% Tween-20). Gills were then incubated in blocking buffer (PBS-T, 1% dimethyl sulfoxide, and 5% donkey serum [Gibco, Carlsbad, Calif]) for 30 minutes, followed by incubation with 1:1000 polyclonal chicken anti–green fluorescent protein (GFP; ab13970; Abcam, Cambridge, United Kingdom) in blocking buffer at 4°C overnight. Gills were rinsed 4 times in PBS-T and incubated in 1:500 polyclonal donkey anti-chicken−Alexa Fluor (AF) 488 (703-545-155; Jackson ImmunoResearch, West Grove, Pa) in blocking buffer for 4 hours at room temperature (RT). Gills were rinsed in PBS-T and PBS before incubating in DRAQ5 (1:1000; Thermo Fisher) and rinsing further in PBS-T.

Gills were imaged on a Leica SP5 inverted confocal microscope (Leica, Wetzlar, Germany), and images were processed with ImageJ software (National Institutes of Health, Bethesda, Md). Cells were counted by using Icy software in the first 20 lamellae from the arch of each undamaged or unobstructed filament. Counts were performed in a blinded manner.

#### Immunohistochemistry of gillsorption

After application on the gills, SAM was fixed in 4% paraformaldehyde (Sigma) for 1 hour at RT. After briefly washing in PBS and deionized water, the SAM was placed in acetone at −20°C for 5 minutes, followed by brief washes with deionized water and PBS-T (PBS, 0.05% Triton-X, and 0.05% Tween-20) and blocking with 5% goat or 5% donkey serum (Sigma) in PBS-T plus 1% dimethyl sulfoxide for 30 minutes at RT. The SAM was incubated with primary antibodies (1:1000, rabbit anti-GFP, A-11122; Life Technologies), mouse anti-cytokeratin antibody (1:100, MA1-82041; Thermo Scientific), and rabbit anti-zebrafish L-plastin antibody (1:500) in blocking solution for 1 hour at RT, followed by washes in PBS-T and incubation in secondary antibodies (donkey anti-rabbit−AF488; 1:500, A-21206; Life Technologies), donkey anti-rabbit−AF555 (1:500, A-21428; Life Technologies), and goat anti-mouse-IgG−AF633 (1:1000, A21052; Life Technologies) for 30 minutes at RT, followed by washes in PBS-T and PBS before incubation in DRAQ5 (1:1000, 62254; Thermo Fisher Scientific) in PBS for 15 minutes at RT. Imaging was performed with an inverted Leica SP5 confocal microscope, and image processing was carried out with Fiji software.

### Statistics

All statistical analysis was carried out with GraphPad Prism 6.0 software (GraphPad Software, La Jolla, Calif). Normality distribution was tested with the D'Agostino–Pearson omnibus test. When comparing 2 groups, unpaired 2-tailed *t* tests (followed by the Welch correction test for nonequal SDs) and Mann-Whitney tests were used for parametric and nonparametric data sets, respectively. Two-way ANOVA followed by a Sidak multiple comparison test was used for time-course data sets. *P* values of less than .05 were deemed statistically significant. For human studies, paired *t* tests of area under the curve (AUC) of log_10_-transformed values between 0 to 8 hours were initially calculated for each mediator in individual subjects (n = 9) after nasal challenge with saline and R848. Subsequently, AUC values were compared between groups by using a paired *t* test. The baseline parameter for AUC was set at the lower limit of detection for each mediator.
